# Wind estimation based on thermal soaring of birds

**DOI:** 10.1002/ece3.2585

**Published:** 2016-11-15

**Authors:** Rolf Weinzierl, Gil Bohrer, Bart Kranstauber, Wolfgang Fiedler, Martin Wikelski, Andrea Flack

**Affiliations:** ^1^ Department of Migration and Immuno‐EcologyMax Planck Institute for OrnithologyRadolfzellGermany; ^2^ Department of BiologyUniversity of KonstanzKonstanzGermany; ^3^ Department of Civil, Environmental and Geodetic EngineeringThe Ohio State UniversityColumbusOHUSA

**Keywords:** animal movement, environmental drivers, meteorological measurements, migration

## Abstract

The flight performance of birds is strongly affected by the dynamic state of the atmosphere at the birds' locations. Studies of flight and its impact on the movement ecology of birds must consider the wind to help us understand aerodynamics and bird flight strategies. Here, we introduce a systematic approach to evaluate wind speed and direction from the high‐frequency GPS recordings from bird‐borne tags during thermalling flight. Our method assumes that a fixed horizontal mean wind speed during a short (18 seconds, 19 GPS fixes) flight segment with a constant turn angle along a closed loop, characteristic of thermalling flight, will generate a fixed drift for each consequent location. We use a maximum‐likelihood approach to estimate that drift and to determine the wind and airspeeds at the birds' flight locations. We also provide error estimates for these GPS‐derived wind speed estimates. We validate our approach by comparing its wind estimates with the mid‐resolution weather reanalysis data from ECMWF, and by examining independent wind estimates from pairs of birds in a large dataset of GPS‐tagged migrating storks that were flying in close proximity. Our approach provides accurate and unbiased observations of wind speed and additional detailed information on vertical winds and uplift structure. These precise measurements are otherwise rare and hard to obtain and will broaden our understanding of atmospheric conditions, flight aerodynamics, and bird flight strategies. With an increasing number of GPS‐tracked animals, we may soon be able to use birds to inform us about the atmosphere they are flying through and thus improve future ecological and environmental studies.

## 1 Introduction

The world that animals traverse is constantly changing. Whether it is ocean currents, vegetation greenness, or wind patterns, these dynamics of the environment shape animal movement decisions at global and regional scales (Berthold, 2001; Nathan et al., 2008). A variety of studies explore the interaction between the environment and movement decisions using large‐scale remote sensing, climatic, and land use datasets (Bartlam‐Brooks, Beck, Bohrer, & Harris, 2013; Bohrer, Beck, Ngene, Skidmore, & Douglas‐Hamilton, 2014; Bohrer et al., 2012; Dodge et al., 2014; Dragon, Monestiez, Bar‐Hen, & Guinet, 2010; Sapir et al., 2011). Flying animals in particular can increase their ground speed, or reduce travel distance and energetic costs by responding to the changing atmosphere (Chapman et al., 2010; Deppe et al., 2015; Flack et al., 2016; Gill et al., 2009; Kranstauber, Weinzierl, Wikelski, & Safi, 2015; Schmaljohann, Liechti, & Bruderer, 2009). In particular, large, long‐distance migrants have served as model systems to explore the relationship between flight performance and weather conditions (Chevallier et al., 2010; Klaassen, Strandberg, Hake, & Alerstam, 2008; Shamoun‐Baranes et al., 2006; Vardanis, Klaassen, Strandberg, & Alerstam, 2011).

**Figure 1:**
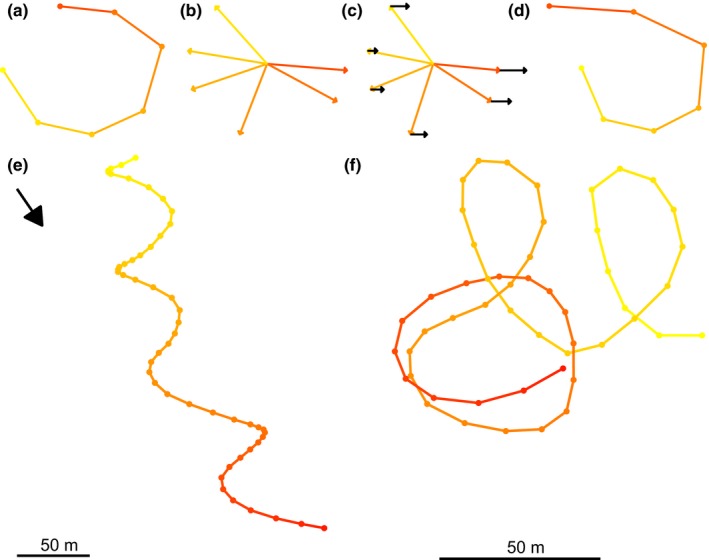
Airspeed, wind speed, and the resulting ground speed derived from a GPS track. (a) In idealized windless conditions, the track of a bird circling through a thermal column, as illustrated . (b) The vectors that connect subsequent positions correspond to the individual movement steps of the bird, or, if we divide it by the time for each step, they represent the bird's airspeed vector. (c) Side wind (black vectors) displaces the bird in each movement step. (d) When combining the bird's movement vectors (airspeed) and the wind vectors (wind speed), we obtain the movement steps of a bird flying under the influence of side wind (ground speed). (e) An observed GPS track (the stork “Lucky.” Sept 1, 2014, 3 p.m.) of a stork, which is displaced by wind while circling. (f) The same track compensated for wind by subtracting a constant wind displacement vector per circle. In all panels, color gradient (yellow to red) indicates time from the beginning of the thermalling event

Here, we introduce a method to estimate wind speed and direction from thermalling behavior of birds. We apply and test it using freely migrating white storks (*Ciconia ciconia*). Thermalling refers to a stereotypic flight maneuver during which birds gain altitude by circling in columns of warm rising air (i.e., “thermals”). The energy gained from climbing thermals depends on the strength and depth of the available thermal uplift (Shamoun‐Baranes, Leshem, Yom‐Tov, & Liechti, 2003). When examining a GPS track, the records represent the sum of the animal movement vector (heading and speed) and the wind vector (Richardson, 1990). In a hypothetical wind‐free environment, high‐resolution records of thermalling behavior will describe short track segments in the shape of closed loops representing the bird's movement relative to the air. In real‐world environments, wind adds a component to the movement vectors and thus distorts these closed loops in the resulting GPS track that depicts the movement relative to the ground (Figure 1). Using these distorted ground‐movement tracks, it is possible to reconstruct both the air movements of birds and the movements of the air itself, that is, the wind. Previous studies have used patterns of thermal soaring to estimate lateral winds displacing birds and gliders (Ákos, Nagy, & Vicsek, 2008; Lerch, 2004; Treep et al., 2016). Here, we further develop this approach in a systematic fashion: We use maximum‐likelihood optimization to minimize the error in wind estimate over short segments representing individual thermal loops; we derive and validate error estimates; follow explicit assumptions about the bird's flight patterns and identify their potential effects on the error or bias of the resulting wind estimate; and point out how our method can be generalized to other behavioral patterns and tracking technologies. We use a large tracking study of storks and data from an atmospheric reanalysis to validate our approach. Finally, we demonstrate that the resulting observations can be useful both for studying the structure of the atmosphere and the flight behavior of storks.

## 2 Material and Methods

### 2.1 Wind estimation theory—general approach

Consider a bird flying horizontally at a constant autocorrelated airspeed in a spatially uniform and temporally constant wind field. The instantaneous ground speed of such a bird depends on its heading relative to the wind. Thus, wind causes variation in ground speed, corresponding with the changes in heading. For example, flight at constant airspeed at a direction directly opposite to the wind will result in lower ground speed compared with flight in the same airspeed but along the wind direction. Hence, theoretically, if ground speed is known and there are sufficient changes in flight heading (but not flight airspeed), we can determine the wind speed and direction from the bird's flight track. In the example above, the wind velocity component in the axis parallel to the flight can be determined by simply subtracting the ground speeds of the two flight tracks in opposing directions. More generally, if we can assume that over a short track segment both airspeed and wind speed are constant, then changes in flight heading will generate variation in the apparent ground speed. We can determine the wind speed as the vector that when subtracted from each ground speed vector will result in constant airspeed (Figure 1). In reality, however, air and wind speed are not constant, and location and speed measurements recorded through GPS include an error. Therefore, it is impossible to accurately determine wind speed from a single GPS measurement. Nevertheless, here we show that it can be approximated using a maximum‐likelihood approach, which minimizes the residual variance of speed over short track segments during thermalling.

To do so, we assume that during thermalling a flying stork tries to move in a circle with a constant airspeed. While it may get pushed away from that constant circling speed by small‐scale turbulent air movements, it will gradually return to it.

### 2.2 Formulation

We define $G=\left[\vec{g_{1}^{\prime }},\ldots ,\vec{g_{n}^{\prime }}\right]$ as a sample of an observed sequence of GPS instantaneous ground speed vectors measured on a flying bird at a constant sampling rate Δ*t* (In this study, we used Δ*t* = 1 [s]) and locations *i*. The observed ground speed vectors consist of *x*‐ and *y*‐components, $\vec{g_{j}^{\prime }}=\left(g_{i,x}^{\prime },g_{i,y}^{\prime }\right)$, representing the locations along the east–west and north–south directions, respectively. We assume that $\vec{g_{i}^{\prime }}$ include some independent measurement errors that follow a circularly symmetric bivariate normal distribution with variance $\sigma_{g}^{2}$. Thus, the true ground speed vectors are defined as: $\vec{g_{i}}=\vec{g_{i}^{\prime }}-N\left(\sigma_{g}^{2}I\right)$, where *I* is the 2 × 2 identity matrix. We further assume a constant horizontal wind vector, $\vec{w}=\left(w_{x},w_{y}\right)$. By definition, the bird's true air‐speed vector, $\vec{a_{i}}=\left(a_{i,x},a_{i,y}\right)$ is:

(1) $$\vec{a_{i}}\equiv \left(\vec{g_{i}}-\vec{w}\right).$$

The scalar airspeed, *a*_*i*_, is defined using the vector‐length operator, that is, $a_{i}=\left\|\vec{a_{i}}\right\|$. We model *a*_*i*_ as a first‐order autoregressive process, AR(1), with mean, *a*, representing the bird's assumed constant airspeed, and unexplained and small‐scale variance due to turbulence, $\sigma_{a}^{2}$, such that $a_{i}\;=\;\left(1-\phi \right)a\;+\;\phi a_{i-1}\;+\;N\left(\sigma_{a}^{2}\right)$, where *N* is a mean‐zero Gaussian random distribution and ϕ (0 ≤ ϕ ≤ 1) is an autocorrelation coefficient. Substituting the observed ground speed vector into the definition of airspeed, we obtain:

(2) $$\vec{a_{i}}=\vec{g_{i}^{\prime }}-N\left(\sigma_{g}^{2}I\right)-\vec{w}$$

By reorganizing equation (2) and assuming that the GPS error σ_*g*_ is small relative to *a*_*i*_, we obtain the following approximation for airspeed, $\tilde{a_{i}}:$

(3) $$a_{i}=\|\left(\vec{g_{i}^{\prime }}-\vec{w}\right)-N\left(\sigma_{g}^{2}I\right))\|\approx \tilde{a_{i}}+N\left(\sigma_{g}^{2}\right).$$

Thus, we can approximate the true airspeed by $\tilde{a_{i}}$, an AR(1) process with mean *a*, unexplained variance $\sigma^{2}\equiv \sigma_{a}^{2}+\sigma_{g}^{2}\left(1-\phi^{2}\right)$, and autocorrelation coefficient, ϕ, such that

(4) $$\left[\begin{array}{cc} & \tilde{a_{i}}=\left(1-\phi \right)a+\phi \tilde{a_{i-1}}+N\left(\sigma^{2}\right)\\ & \text{var}\left(\tilde{a_{i}}\right)=\frac{\sigma^{2}}{1-\phi^{2}} \end{array}\right.$$

We can derive the negative log likelihood of observing the sequence of GPS ground speed vectors, *G*, given wind $\vec{w}$ as

(5) $$\begin{array}{cc} l(G,\vec{w}) & \;\;=-\text{log}\left(P\left({\tilde{\alpha }}_{1}\right))\prod_{i=2}^{n}P\left({\tilde{a}}_{i}|{\tilde{a}}_{i-1}\right)\right)\\ & =\frac{1}{2}\left\{\text{nlog}\left(\sigma^{2}\right)-\text{log}\left(1-\phi^{2}\right)\right.\\ & \left.+\frac{1}{\sigma^{2}}\left[{\left({\tilde{\alpha }}_{1}-a\right)}^{2}\left(1-\phi^{2}\right)+\sum_{i=2}^{n}{\left({\tilde{\alpha }}_{i}-\left[\left(1-\phi \right)a+\phi {\tilde{\alpha }}_{i-1}\right]\right)}^{2}\right]\right\} \end{array}$$

where constant terms were omitted. By setting the derivative of the likelihood function with respect to the wind speed vector approaching zero, we obtain the maximum‐likelihood estimator for mean airspeed:

(6) $$\hat{a}=\frac{{\tilde{\alpha }}_{1}+\left(1-\phi \right)\sum_{i=2}^{n-1}{\tilde{\alpha }}_{i}+{\tilde{\alpha }}_{n}}{1+(n-2)(1-\phi )+1}=\frac{\|\vec{g_{i}}-\vec{w}\|+\left(1-\phi \right)\sum_{i=2}^{n-1}\|\vec{g_{i}}-\vec{w}\left\|+\right\|\vec{g_{n}}-\vec{w}\|}{1+(n-2)(1-\phi )+1}$$

Using this estimator and applying Bessel's correction for sample size, that is, multiplying by $\left[n/\left(n-1\right)\right]$, and assuming that $\tilde{a}\approx a$, we get an estimator for the variance term in equation (5):

(7) $$\begin{array}{cc} s^{2}\left(G,\vec{w}\right)\;\; & \equiv \frac{1}{n-1}\left[{\left({\tilde{\alpha }}_{1}-\hat{a}\right)}^{2}\left(1-\phi^{2}\right)+\sum_{i=1}^{n}{\left({\tilde{\alpha }}_{i}-\left[\left(1-\phi \right)\hat{a}+\phi \tilde{\alpha_{i-1}}\right]\right)}^{2}\right]\\ & =\frac{1}{n-1}\left[{\left(\|\vec{g_{i}}-\vec{w}\|-\hat{a}\right)}^{2}\left(1-\phi^{2}\right)\right.\\ & \left.+\sum_{i=2}^{n}\left(\right\|\vec{g_{i}}-\vec{w}\|-[\left(1-\phi \right)\hat{a}+\phi \|\vec{g_{i}}-\vec{w}{\left\|]\right)}^{2}\right] \end{array}$$

and obtain:

(8) $$l\left(G,\vec{w}\right)=\frac{1}{2}\left\{\text{nlog}\left(\sigma^{2}\right)-\text{log}\left(1-\phi^{2}\right)+\frac{n}{\sigma^{2}}s^{2}\left(G,\vec{w}\right)\right\}$$

Because the first two terms and the factor *n*/σ^2^ are independent of the wind speed vector, we can calculate the likelihood estimate for the unknown wind vector by minimizing the unexplained variance in airspeed $s^{2}\left(G,\vec{w}\right)$:

(9) $$\hat{w}=\underset{\vec{w}}{\text{arg min}}\left[s^{2}\left(G,\vec{w}\right)\right].$$

By re‐inserting the estimated wind vector $\hat{w}$ into the expression $s^{2}\left(G,\vec{w}\right)$, we get an estimate for the variance in airspeed from the observed variance in the wind speed estimates:

(10) $${\hat{\sigma }}^{2}=s^{2}\left[G,\hat{w}\right]$$

which we insert into the expression for the likelihood function (equation (8)) to obtain

(11) $$l\left(G,\vec{w}\right)=\frac{1}{2}\left\{\text{nlog}\left({\hat{\sigma }}^{2}\right)-\text{log}\left(1-\phi^{2}\right)+\frac{n}{{\hat{\sigma }}^{2}}s^{2}\left(G,\vec{w}\right)\right\}$$

By taking the inverse of the Hessian of this expression with respect to $\hat{w}$, we get the covariance matrix which characterizes the error of the wind estimate $\hat{w}$:

(12) $$\sum ={\left(\frac{n}{2{\hat{\sigma }}^{2}}\frac{d^{2}}{d{\vec{w}}^{2}}s^{2}\left(G,\hat{w}\right)\right)}^{-1}$$

### 2.3 Calculation of flight characteristics

Utilizing the wind estimates (equation (9)), we can further calculate various properties of flight and airspeed. In order to calculate those properties of the *k* ^th^ GPS fix, we choose an integer *m* and consider a track segment of length *n = 2m* + 1 centered at that *k* ^th^ fix ($G=[{\vec{g}}_{k-m},\ldots ,\;{\vec{g}}_{k},\ldots ,\;{\vec{g}}_{k+m}]$). All angles are in radians and normalized to $\left[-\pi ,\pi \right]$.

For each track segment, we obtain mean airspeed $\hat{a}$ (equation (6)), by setting $\vec{w}=\hat{w}$; the magnitude of the unexplained deviation in airspeed is calculated as $\sqrt{s^{2}\left(G,\hat{w}\right)}$ (equation (10)). Also for each segment around point *k*, mean vertical ground speed, $g_{z_{k}}$, is calculated as

(13) $$g_{z_{k}}=\frac{\left(z_{k+m}-z_{k-m}\right)}{\left(n-1\right)\Delta t}$$

where *z*_*k*_ is the GPS height at point *k*. Change in heading between two consecutive GPS fixes, Δθ_*i*_, is defined as the angle between the airspeed vectors $\left(\vec{g_{i}}-\hat{w}\right)$ and $\left({\vec{g}}_{i+1}-\hat{w}\right)$; the cumulative change in heading can then be defined as $\Delta \theta_{\mathrm{cum}}\equiv \sum_{i=k-n}^{k+n-1}\Delta \theta_{i}$. Assuming that the bird is flying in a perfect circle at constant airspeed, we calculate the mean angular rate in radians, ω, the circle radius, *r*, and the time per full circle, Δ*t*_*c*_, are:

(14) $$\omega =\frac{\Delta \theta_{\mathrm{cum}}}{\Delta t_{\mathrm{cum}}};\;r=\frac{\hat{a}}{\omega };\;\Delta t_{c}=\frac{2\pi }{\omega }$$

Using the fact that in a balanced turn the centripetal acceleration is $\hat{a}\omega =\left(L/m\right)\sin\left(\beta \right)$ and the gravitational acceleration g=(L/m)cos(β), where *L* is the lift, *m* the bird mass, and β the banking angle, we obtain estimates for the banking angle:

(15) $$\beta =\tan^{-1}\left(\frac{\hat{a}\omega }{g}\right)$$

and the lift acceleration:

(16) $$\frac{L}{m}=\sqrt{g^{2}+{\hat{a}}^{2}\omega^{2}}$$

We use the estimated airspeed $\hat{a}$, the lift acceleration *L*/m, and default aerodynamic properties of storks (Eder, Fiedler, & Neuhäuser, 2015) to calculate the drag acting on the bird and its sink rate relative to the air. We define drag coefficient, *C*_*D*_, induced drag coefficient, *C*_*D,i*_, the noninduced drag coefficient, *C*_*D,*ni_, lift coefficient, *C*_*L*_, and total drag, *D*, following (Eder et al., 2015):

(17) $$C_{D}=C_{D,i}+C_{D,\mathrm{ni}},$$

(18) $$C_{D,i}=\frac{KC_{L}^{2}}{\pi A_{R}},$$

(19) $$C_{L}=L\frac{2}{\rho {\hat{a}}^{2}S},$$

(20) $$D=C_{D}\frac{\rho {\hat{a}}^{2}S}{2},$$

where *K* is the induced drag factor, *A*_*R*_ is the wing aspect ratio, ρ the air density, and *S* the wing area. To obtain a rough estimate of total drag, *D* (equation (20), Pennycuick, 2008), we used default parameters for soaring storks from (Eder et al., 2015): *C*_*D*,ni_ = 0.033, *K* = 0.81, *S *=* *0.57 [m/s^2^], *A*_*R*_ = 7.21, m = 3.63 [kg] and ρ = 1.15 [m/s^2^].

The loss rate of kinetic energy caused by drag is *E*_kin_ = $D\hat{a}$. This means that a bird maintaining constant airspeed has to gain kinetic energy from potential energy and consequently sinks relative to the surrounding air mass at a rate of

(21) $${\hat{a}}_{z}=\frac{D\hat{a}}{mg_{a}}$$

where *g*_*a*_ = 9.81 [m/s^2^] is Earth's gravity and mg_*a*_ is the bird's weight. By subtracting *a*_*z*_ from the mean vertical ground speed, we yield an estimate of the mean vertical wind speed, or thermal strength, ${\hat{w}}_{z}={\hat{a}}_{z}-g_{z}^{\prime }$.

Wind estimation was performed in Java^™^ using the Nelder–Mead Simplex optimization algorithm (Nelder & Mead, 1965) and the numerical derivation procedure implemented in the apache‐commons‐math‐3 package, version 3.4.1. All other statistics were calculated in R, version 3.2.0 (https://www.r-project.org/).

A library of functions in R that calculates wind speed, thermal strength, uplift strength, and thermalling circle radius using our newly introduced method is available through the R package “moveWindSpeed” (https://cran.r-project.org/package=moveWindSpeed), which supplements the R “move” package (https://cran.r-project.org/web/packages/move/index.html).

### 2.4 Data selection

Our wind estimation method depends on changes in flight heading. In theory, it is not limited to circular thermal soaring, but will work for any observation period during which a consecutive sequence of more than one point represents a movement pattern with a constant turn angle.

Here we leveraged on the fact that during thermalling flight we can assume that birds turn at a constant rate at least over a single thermalling “circle.” We therefore restricted the analysis to circling events in a thermal, that is, birds flying in either closed or stretched circles (illustrated in Figure 1) while having a positive mean upward vertical velocity. First, we identified circling events using a preliminary wind estimate that ignores the effects of temporal autocorrelation (ϕ = 0). We selected sequences of 19 subsequent GPS points sampled at 1 [Hz] (18 [s] is the 95% quantile of observed time per full circle, 16.32 [s], plus a small margin. See Table 1). We then determined the turning angles between these subsequently wind‐compensated speed vectors. A track segment was classified as a circling event when all angles had the same sign and the sums of angles before and after the central point each exceeded 180°. After removing sequences that were overlapping in time, we obtained 24,495 circling events from 60 storks, representing 170 hr of circling time.

**Table 1:** Summary statistics (columns showing different quantiles) of flight characteristics, determined from thermalling flight (*n* = 24,495 circling events)

	5%	25%	50%	75%	95%
Wind speed [m/s]	0.50	1.38	2.47	4.18	7.21
Mean airspeed [m/s]	8.32	8.93	9.37	9.83	10.56
Standard deviation of airspeed [m/s]	0.26	0.36	0.46	0.59	0.84
Mean vertical speed over ground [m/s]	0.04	0.48	0.92	1.49	2.52
Mean sink speed relative to air [m]	0.71	0.73	0.75	0.78	0.83
Circling radius [m]	15.71	18.32	20.31	22.38	25.48
Time per full circle [s]	10.98	12.49	13.65	14.79	16.32
Banking angle [degrees]	19.75	21.94	23.76	25.88	29.14

Next, to determine a nontrivial autocorrelation coefficient ϕ of those 24,495 circling events, we calculated the log likelihood of ϕ as the sum of the maximum log likelihoods and maximized over ϕ. We obtained an estimate of $\hat{\phi }$ = 0.47. The average log likelihood across all circling events for $\phi =\hat{\phi }$ was 1.94 points higher than for ϕ = 0. Using $\phi =\hat{\phi }$ (instead of ϕ = 0), we re‐evaluated our classification of circling events, resulting in 24,495 circling events (i.e., 20 previously classified circling events were rejected). Finally, we estimated wind speed, airspeed, vertical speed, circling radius, time per full circle, and banking angle for each circle (see Methods for details and Table 1 for a summary of the results).

### 2.5 Validation of wind estimates

Our wind estimate depends on the assumption of independent, random, small‐scale airspeed variation. However, if this assumption is false, our estimates would be biased. For example, airspeed variations within a thermalling circle may become highly autocorrelated in a way that will bias our estimate if the bird varied its airspeed within each circle depending on its flight direction relative to the wind. To evaluate the accuracy of our estimates and the extent to which it was affected by variation of flight behavior within a thermalling circle, we compared the estimated wind conditions of two birds flying closely together within the same thermal. Next, we checked for behavioral adjustments, which could violate the assumptions of our model (e.g., variation of flight speed, or banking angle as a function of direction within each thermalling circle) by looking at the wind properties of different sections of the same circle. Finally, we compared our estimates with wind estimates from a weather reanalysis dataset.

#### 2.5.1 Two birds soar in the same thermal

**Figure 2:**
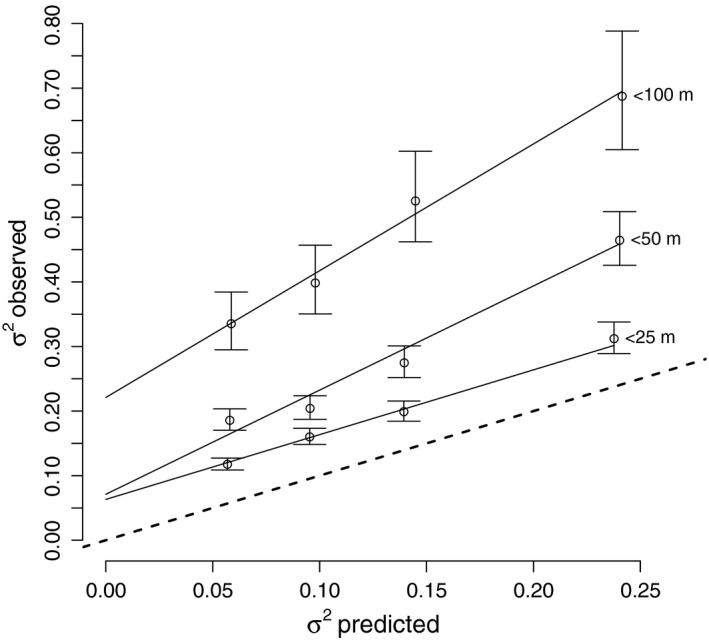
Pairs of birds circling nearby (assumingly in the same thermal) in opposite directions were used to evaluate the error estimate of our wind estimation method. Cocircling birds were classified into one of three groups based on distance class (<25, <50, <100 m, indicated at the right of each curve). Next, we split the partial deviances from each distance category into four groups of equal size based on the estimated variances (equation (10)) and calculate the mean observed and predicted variances, σ^2^. The observed variance was estimated from the variation between the paired wind estimates of birds within each group. Error bars mark the 95% confidence interval

Given the lack of direct independent measurements of wind speed at the birds' locations, we compared pairs of independent wind estimated from two birds flying nearby to evaluate the accuracy of our approach. Consider wind estimates ${\hat{w}}_{1}$ and ${\hat{w}}_{2}$ derived from the tracks of two birds (bird_1_ and bird_2_, respectively) circling at close proximity to each other in the same thermal column. We assume that they encounter the same true mean wind speed. If we further assume that our wind estimates are statistically independent and their corresponding error estimates Σ_1_ and Σ_2_ are correct, their difference vector $\left({\hat{w}}_{2}-{\hat{w}}_{1}\right)$ has a distribution $\left(N\left(\Sigma_{1}+\Sigma_{2}\right)+T\right)$, where *T* is a random variable accounting for turbulence‐driven difference in wind speed. We assume that *T* is circularly symmetric bivariate with mean zero and variance $\sigma_{T}^{2}$. Therefore, (${\hat{w}}_{2}-{\hat{w}}_{1}$) is bivariate normally distributed with mean zero and variance $\Sigma_{1}+\Sigma_{2}+I\sigma_{T}^{2}$ and can be decomposed into two independent random variables *d*_major_, *d*_minor_ along the eigenaxes of the error covariance matrix Σ_1_ + Σ_2_, with distributions $N\left(\sigma_{\mathrm{major}}^{2}+\sigma_{T}^{2}\right)$ and $N\left(\sigma_{\mathrm{minor}}^{2}+\sigma_{T}^{2}\right)$, respectively, where $\sigma_{\mathrm{major}}^{2}$ and $\sigma_{\mathrm{minor}}^{2}$ are the eigenvalues of the covariance matrix Σ_1_ + Σ_2_. In other words, the difference vector ${\hat{w}}_{2}-{\hat{w}}_{1}$ can be decomposed into two observed “partial deviances” $\left\{d_{\mathrm{major}},d_{\mathrm{minor}}\right\}$ with variances $\left\{\left(\sigma_{\mathrm{major}}^{2}+\sigma_{T}^{2}\right),\left(\sigma_{\mathrm{minor}}^{2}+\sigma_{T}^{2}\right)\right\}$. Thus, a set of *n* independent pairs of estimates ${\left\{{\hat{w}}_{1},{\hat{w}}_{2}\right\}}_{i}$ can be represented as a set of 2n independent partial deviances *d*_*i*_ with associated distribution $N(\sigma_{\mathrm{est},i}^{2}+\sigma_{T,i}^{2})$. We excluded from the analysis individual circling events for which the estimated variance in airspeed (equation (10)) was larger than 1.0 [m/s] because they represent a poor model fit and unreliable wind speed estimates. We classified the final dataset of pairwise circling events into three categories defined by their distances to each other: (1) 0–25 m; (2) 25–50 m; and (3) 50–100 m. This allowed us to quantify the effect of interindividual distances on the differences in our wind speed estimates. We selected pairs randomly from each distance category, such that any stork was part of only one pair at any time point. For each pair, we calculated the difference vector between the two wind estimates and decomposed it into its x‐ and y‐components with their corresponding variance estimates. Thus, we represented *n* independent pairs of wind estimates as 2n independent partial deviances. Next, we split the partial deviances from each distance category into four groups of equal size based on the estimated variances (equation (10)) and for each of these groups calculated the mean observed variance and the corresponding 95% confidence interval. For each distance category, we fitted a linear function to the relationship between observed and predicted variance (Figure 2).

#### 2.5.2 Variation of airspeed within a thermalling circle

Behaviorally driven variation of flight speed and direction during thermalling flight could violate our assumption of random error distribution around the mean wind speed. This may occur, for example, if a bird's airspeed was higher in the upwind section, compared to the downwind section within each thermalling circle. To estimate the potential sensitivity of our method to such behaviorally driven bias, we considered a bird that changed its heading at a constant rate *ω* and varies its airspeed *a* according to its orientation relative to the wind direction *δ*, with a constant speed component *a*_0_ and a speed amplitude of *a*_*c*_:

(22) $$a\left(\delta \right)=a_{0}-\frac{a_{c}}{2}\cos\delta .$$

For such a bird the airspeed component in the wind direction, a(δ)cosδ could be averaged over the entire circle using an integral average approach as:

(23) $$\begin{array}{cc} \frac{1}{2\pi }\int_{-\pi }^{\pi }a\left(\delta \right)\cos\delta & =\frac{1}{2\pi }\int_{-\pi }^{\pi }a_{0}\cos\delta -\frac{a_{c}}{2}\cos^{2}\delta \\ & =\frac{-a_{c}}{4\pi }{\left[\frac{1}{2}\delta +\frac{1}{4}\sin2\delta \right]}_{-\pi }^{\pi }=\frac{-a_{c}}{4} \end{array}$$

This biases the wind estimate in that direction by approximately ‐*a*_*c*_/2.

Periodic variation in airspeed while gliding would also cause changes in vertical speed, as airspeed variation corresponds to a transformation of potential to kinetic energy (i.e., height vs. speed). According to our model, we can make the following statements: For a circling time of $13\left[s\right]\approx 4\pi \left[s\right]$, the maximal and minimal rates of airspeed change would be $\pm \frac{1}{2}a_{c}\omega \approx \pm \frac{1}{4}a_{c}$; and while flying at an airspeed of 10[m/s], a bird would lose approximately 1 [m] of height to increase its airspeed by 1 [m/s]. Thus, biasing the wind estimate by an amount of *a*_*c*_/2 through periodic variation in airspeed within the thermalling circle would cause a variation in vertical speed with about the same amplitude ≈ *a*_*c*_/2. Here, we used our observations of white storks to test whether such variation in vertical airspeed had occurred and indicated that our estimates might have been biased.

#### 2.5.3 Comparison with wind estimate from weather reanalysis data

There were no direct wind measurements that could be used for independent evaluation of the accuracy of our wind estimation approach, as airborne wind measurement providing data at high elevations above ground are not common. Nonetheless, weather reanalysis models use large datasets of balloon, satellite, and ground station observations to consistently interpolate wind speed in space (horizontally throughout the models' region, and vertically across elevations) and time. Reanalysis models' data are widely accepted as the best available substitute for direct weather observations and are used globally for weather prediction. We used the Environmental‐Data Automated Track Annotation server to obtain wind speed data from The European Centre for Medium‐Range Weather Forecasts (ECMWF) Global ERA‐Interim Daily Mid‐Resolution Reanalysis (see details in section *ENVIRONMENTAL DATA ANNOTATION* below) for 23,921 disjoint circling events. We calculated the hourly average wind speed and direction for each bird with observed circling events to prevent pseudoreplication. We calculated these means for both observed wind from the circling data and for annotated wind speed estimates from the ECMWF dataset. We used the correlation between our wind speed estimates and ECMWF's to evaluate the accuracy of our wind estimates. We used vector correlation (Crosby, Breaker, & Gemmill, 1993) to check for agreement between ECMWF and track‐estimated wind. The correlation's significance was calculated using simulations using 1,00,000 random resamples.

### 2.6 Study species and track measurements

We equipped juvenile white storks (*Ciconia ciconia*) with high‐resolution, solar GSM‐GPS‐ACC loggers. We focused our tracking efforts on a small stork colony in the South of Germany (47°45′10.8″N, 8°56′2.4″E), which consisted of 22 nests. In total, 61 juveniles, the colony's entire offspring, were equipped with high‐resolution, solar GSM‐GPS‐ACC loggers (e‐obs GmbH; Munich, Germany) 1 week prior to fledging. Birds were fitted with a tag during June and July 2014. High‐frequency GPS measurements were reported for each bird during the fall migration season, until September 2014. Tagging permit number G‐13/28 was issued by Regierungspräsidium Freiburg (Federal State of Baden‐Württemberg, Germany).

The transmitters (weight 54 [g]) were attached using a Teflon–nylon harness (weight ~12 [g]). The total weight of transmitter and harness was 66 [g], corresponding to approximately 2% of the mean body mass of white storks (Creutz, 1985). We recorded GPS locations for 18 [hr] a day (between 4:00 and 22:00 local time at the natal grounds). Each fix consisted of geographic position and elevation in WGS84 coordinates, speed and heading, as well as error estimates for position and speed. GPS speed and heading were converted to Cartesian coordinates. The GPS was set to provide high‐frequency (1 [s]) observations for 5 min. Every 15 min positions have a positional accuracy of ± 3.6 [m] (i.e., when stationary, 50% of fixes remain within a radius of 3.6 [m] within 24 hours). Data were stored onboard the device until downloaded via a UHF radio link from a distance of approximately 300 [m] (Holland, Wikelski, Kümmeth, & Bosque, 2009). The data were then stored in the Movebank database (Kranstauber et al., 2011). The data that were used for this study are part of the “MPOI white stork lifetime tracking” data (Flack et al., 2016) and are available through the Movebank Data Repository (Weinzierl et al., 2017).

### 2.7 Environmental data annotation

We used the Environmental‐Data Automated Track Annotation (Env‐DATA) system (Dodge et al., 2013) to annotate the tracking data with ambient atmospheric observations and with ground elevations. Env‐DATA is a service of Movebank (www.movebank.org, Kranstauber et al., 2011), an open, online system for management, archiving, analysis, and sharing of animal movement data. The European Centre for Medium‐Range Weather Forecasts (ECMWF) Global ERA‐Interim Daily Mid‐Resolution Reanalysis dataset (http://www.ecmwf.int/en/research/climate-reanalysis/era-interim, Dee et al., 2011) was accessed to annotate the tracks with wind velocity. We obtained the wind speed at different pressure levels and interpolated it first horizontally at each pressure level to the bird's location using a bilinear interpolation between the reported wind speeds at the four ECMWF grid locations surrounding the bird. Second, we interpolated vertically between the two pressure levels adjacent to the bird's elevation above ground using a linear interpolation. We assume that given the short distances between the pressure levels and the typical flight height, the linear interpolation would not result in a significant error. We did not use more complex interpolation methods as those require knowledge of the surface roughness and heat flux and would introduce additional error. Ground elevation for each observed track point was obtained from NASA ASTER GDEM dataset (https://asterweb.jpl.nasa.gov/gdem.asp, Tachikawa, Hato, Kaku, & Iwasaki, 2011). The flight elevation was evaluated by converting GPS‐obtained height above ellipsoid to height above sea level and then subtracting the annotated ground elevation.

### 2.8 Implementations

We conduct two implementation studies to showcase our approach's potential to produce meaningful data for studying, analyzing and modeling the flight behavior of birds, and to provide otherwise hard‐to‐obtain observations of the wind conditions throughout the atmospheric boundary layer.

#### 2.8.1 Vertical variation in airspeed in a thermal

Thermals have a limited and relatively small (tens to hundreds of meters) size. Therefore, birds need to bank at an angle that is determined by the radius of the circle and their own flight speed (equation 24) in order to stay in a zone with strong uplift. Identifying the choice of bank angle and flight speed as a function of thermalling radius size, strength, and elevation informs about the bird's flight behavior and could be used to identify its strategy and skill for detecting thermals and maintaining uplift throughout thermal flight at a large range of elevations (e.g., Harel, Horvitz, & Nathan, 2016; Harel, Duriez, et al., 2016; Sherub, Bohrer, Wikelski, & Weinzierl, 2016).

We selected 18,086 disjoint circling events with a mean vertical ground speed above 0.5 [m/s] and calculated the circle radius (equation (14)), banking angle (equation (15)), and lift coefficient (equation (16)). We grouped data according to circle radius (group size = 2 [m], groups with <10 elements omitted). To determine the relationship between airspeed and banking angle, we fitted linear models using quantile regression with τ = 0.5 (median).

#### 2.8.2 Observations of vertical profiles of wind speed and detection of the elevation of the atmospheric boundary‐layer top

Wind measurements at elevations of more than 10 meters above ground (the typical height of a meteorological ground station) are rare, spatially sparse and temporally intermittent, because they can only be conducted only from tall towers, weather balloons, and aircrafts. Here, we show that bird‐borne observations (especially those conducted by several birds flying nearby in the same environment) can be used as an alternative source for such information.

We combined the wind estimates from all thermalling individuals in a flock. We defined groups of nearby points (i.e., individual “thermals”) by rasterizing the longitude × latitude plane into squares of 0.1° × 0.1° and the time axis into 15‐minute intervals. We calculated wind speed and thermal uplift and related them to height above ground and averaged these for all birds within each individual “thermal” to produce a vertical profile of wind speed and uplift strength throughout the thermal column.

## 3 Results

### 3.1 Validation of wind estimates

#### 3.1.1 Pairwise comparison of independent estimates

To validate our wind estimates and evaluate their error distributions, we used circling events in which two birds flew in close proximity to each other, assuming that both birds encountered the same true mean wind speed. Each of these pairwise wind estimates is considered as an independent measurement of the same wind. We only evaluated wind estimates of pairs flying in opposite directions (i.e., clockwise and counterclockwise in the same thermal) so as to reduce the influence of behavioral coordination (i.e., following behavior) on the independence of the pair of estimates. These were further classified to three groups according to the distance between the pair of birds. Our results demonstrated that wind estimates differed more when birds were further apart from each other. Also, the observed difference in wind speed estimates grew with the estimated error (Figure 2). For validating the error estimates, we assumed that the results from shortest distance category (Category I, <25 m) minimized the difference in wind conditions encountered by the two birds. Based on the slope and the intercept of the linear relationship between the observed and predicted variation of speeds within pairs, we can assume that two birds encountered slightly different wind conditions (intercept = 0.06), but a slope of 1.0 indicates that the covariance matrices obtained from the maximum‐likelihood estimation were accurate (Figure 2). To get a direction‐independent error estimate, we calculated the 95% CI in the direction with maximum error (i.e., along the major axis of the eigen‐decomposition of each covariance matrix). We found that 95% of all CIs were within ± 0.87[m/s], and 50% within ±0.47[m/s].

#### 3.1.2 Behavioral variation in different sections of a circle

**Figure 3:**
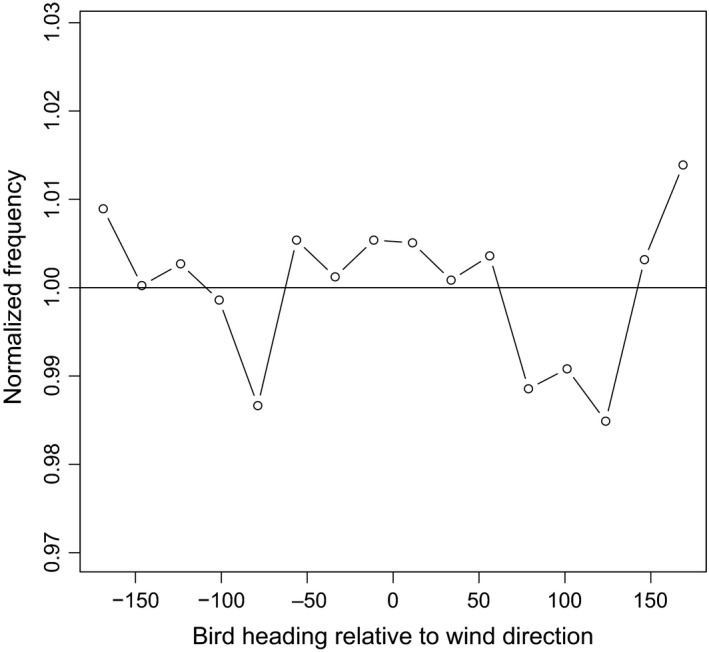
Normalized frequency (mean = 1) of bird heading relative to wind direction [degrees]. The variation around the mean is very small (<|1.5%|) indicating that all directions are sampled equally and, therefore, that the observed storks did not preferentially spend more or less time in the upwind part of the circle relative to downwind (or any other circle segment)

Behaviorally driven variation in flight speed and direction within a circling event might violate our assumption of a random error distribution around the mean wind speed. To determine whether this was the case in our dataset of stork‐borne observations, and to estimate the potential error that may result from such an effect, we analyzed the wind speed estimate as a function of the bird's heading relative to the wind (Figure 3).

If the birds behaved differently in the upwind versus the downwind sides of the thermalling circle, we would expect that the birds consistently spend more time flying at one side of the circle or fly faster when orientated upwind or downwind. However, we did not find evidence for either of these two predictions. Figure 3 shows that the time spent in different orientations varied by less than two percent. Similarly, the mean estimated airspeed deviation from the circle mean changed by less than 0.035 [m/s] (not shown).

#### 3.1.3 Comparison of wind estimate with independent atmospheric data

**Figure 4:**
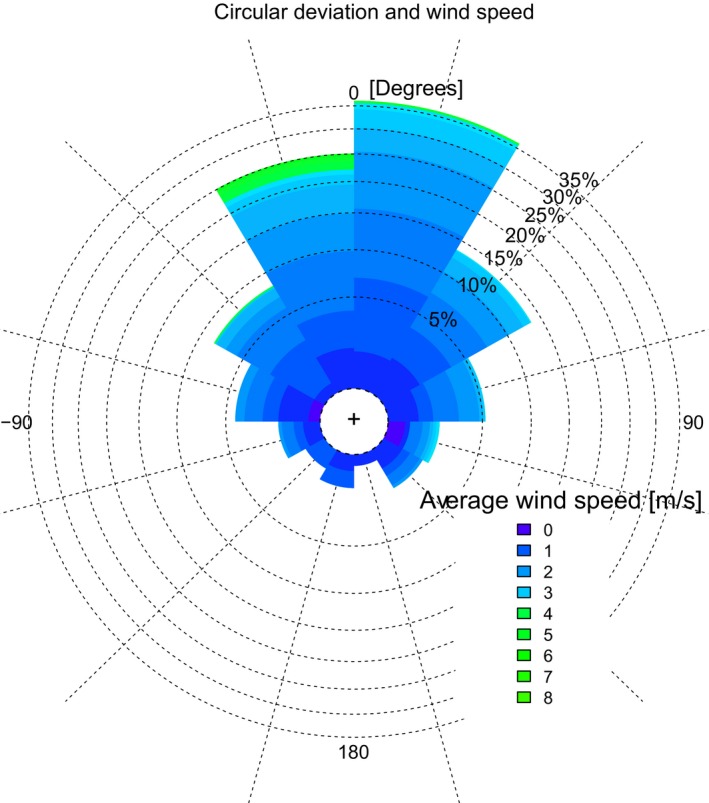
The frequency distribution of the difference between the track‐estimated wind direction and the wind direction from the ECMWF data. Color indicates the averaged wind speed between the ECMWF and track estimates. At low wind speeds, there is low agreement on the wind direction but as soon as the wind speed increases there is a high agreement between the two wind estimate approaches. The number of observations: *n* = 570. Significance *P* < .001

There was a high correlation between track‐estimated and ECMWF wind vectors (Figure 4, *r* ^2^ = .469, *P* < .001). The average magnitude (vector length) of the difference between the two vectors was 1.471. The average ECMWF wind speed was 2.347 [m/s] and the track‐estimated one was 1.913 [m/s]. A linear regression between the ECMWF and track‐estimated wind speeds (intercept = 1.17, slope = 0.62) indicated a systematic bias between the two wind estimates, whereas the track‐observed wind speed tended to be higher than the ECMWF estimate when the wind was strong, and lower when the wind was very weak.

## 4 Discussion

Here, we introduced and tested a new approach that uses short, high‐resolution GPS segments of thermalling birds to obtain estimates of wind speed and direction. By examining a large set of pairs of independent wind estimates from two white storks circling in close proximity in the same thermal, we determined that our approach provided wind estimates with an accuracy of about half a meter per second (Figure 2). A comparison with wind estimates from the ECMWF weather reanalysis dataset showed a systematic bias between the two wind estimates, where ECMWF estimates provided less extreme values (i.e., lower when wind was strong, higher when wind was weak) than the wind observed from the bird tracks. But because the ECMWF provided spatially averaged wind predictions along a gridded space with a resolution of roughly 25 × 25 [km^2^], this type of bias was expected. At such resolution, fast perturbations of the wind were averaged out leading to lower mean wind speed compared to point measurements at a particular spot that may capture such local extremes. Similarly, when the wind was very weak at a particular location and time, averaged predictions might overestimate the actual wind speed. Given that the mean wind field is scale dependent, the consistent bias between the bird‐borne and ECMWF wind estimate did not indicate a problem in any of the datasets, as this bias was related to a mismatch in the scales which these two data sources represent. We therefore claim that a good correlation between the two data sources confirms the quality of both, as it would be very hard to demonstrate such a high fit between two independently estimated datasets if one or both had large random errors.

Recently, Treep et al. (2016) estimated wind properties from tag‐based high‐frequency GPS data of soaring vultures. Although the wind estimation approach by Treep et al. (2016) produces acceptable estimated for wind speed, their approach works under the assumption of constant wind speed and turning angle over two thermalling circles, as it analyses the drift rate from one circle centroid to the next. Our approach relaxes this assumption to a single thermalling circle (and in theory, even less than that) allowing a more extensive use of datasets because it enables the analysis of shorter thermalling events that could not be used by the approach of Treep et al. (2016). Furthermore, our new method demonstrated a more accurate fit to wind direction than previous approaches; and it calculates the variance of the estimate around a circle, which can be used as an error estimate for quality control of the resulting wind estimates.

A good understanding of the relationship between bird movements and meteorology may emerge as a valuable source of needed meteorological information. Measurements of vertical wind profiles are still scarce because they require airborne observations (Shannon, Young, Yates, Fuller, & Seegar, 2002b). The most common of such airborne measurement, conducted by weather balloons (radiosondes), are typically launched only twice a day in designated spots (e.g., airports, meteorological stations). Vertical profiles of wind speed provide important data for flight and weather predictions, and for forcing and evaluation of atmospheric models.

**Figure 5:**
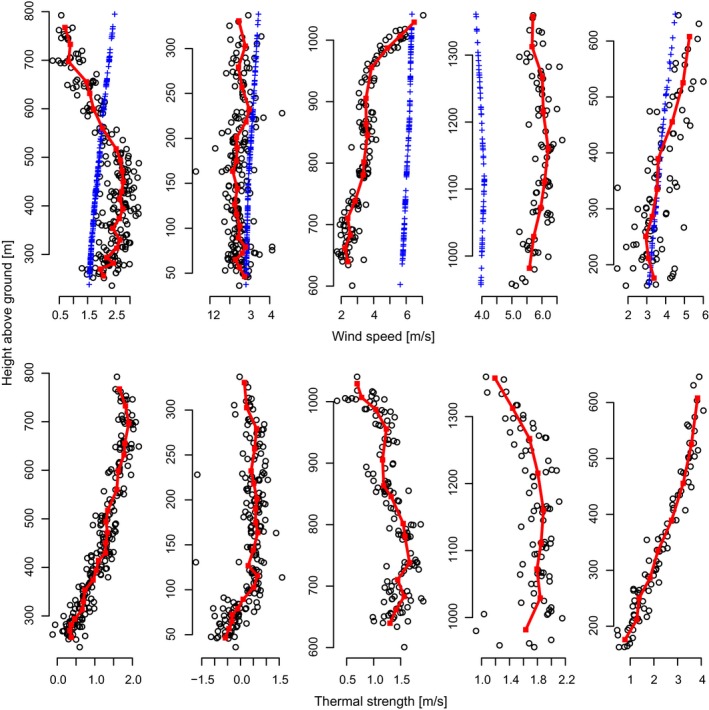
Vertical structure of different thermals: Stork‐track derived wind speed and thermal strength from the same thermal are shown on top of each other. Red lines are smoothed by calculating group averages from 10 subsequent points. Blue crosses in the upper row are wind speeds from ECMWF. Sample sizes (number of thermalling circles) are, from left to right: 214; 192; 131; 81; and 84. Numbers of storks per group: 21; 26; 24; 25; and 12

Using our approach, we demonstrated the potential application by producing a detailed characterization of the vertical profile of wind conditions in a thermal column. Using the storks as sensors, we were able to illustrate different boundary‐layer structures from a selection of thermals (Figure 5). For example, a sharp increase in wind speed at an elevation of 950 [m] above aground, corresponding with a sharp decrease in thermal strength, indicated that the capping inversion at the top of the atmospheric boundary layer was roughly at that elevation (Figure 5, 3^rd^ column). Boundary‐layer height is a critical meteorological variable and is diagnosed by all meteorological models but rarely measured directly. Our result shows that in addition to improving our understanding of atmospheric conditions, this new approach also provides valuable data for the study of flight behavior and properties. A recent study observed that adult griffon vultures adjust their airspeed and heading within a thermalling circle to achieve higher climb rates (Harel, Horvitz, et al., 2016). Because our method assumes a random error distribution around the mean wind speed, such behavioral variation could potentially bias our wind estimate due to an autocorrelated within‐circle error structure. However, our findings show that in white storks this type of behavioral variation within a thermalling circle is small and does not bias our wind speed estimates (Figure 3). Nonetheless, our method is valid for any flight segment with consistent turning angle and is not limited to full thermal circles. Therefore, our assumption of a random error distribution around the mean wind speed could be relaxed from the entire thermalling circle to a predetermined fraction of it (e.g, half a circle) as long as the observation frequency supports at least three wind estimates within a circle section. We recommend that for analyzing thermalling flight of species such as vultures, which were observed to display variation in flight behavior around a thermal, two independent estimates of wind speed—one from the upwind and one from the downwind half of the thermalling circle—should be calculated. Such an approach will also result in two estimates for airspeed. For cases in which the rotation speed of the air column is much weaker than the horizontal wind speed, the wind speed estimates from the opposing side of the thermal are expected to represent the outcome of behavioral flight strategy around the thermal, and will differ in birds that display such behavior.

**Figure 6:**
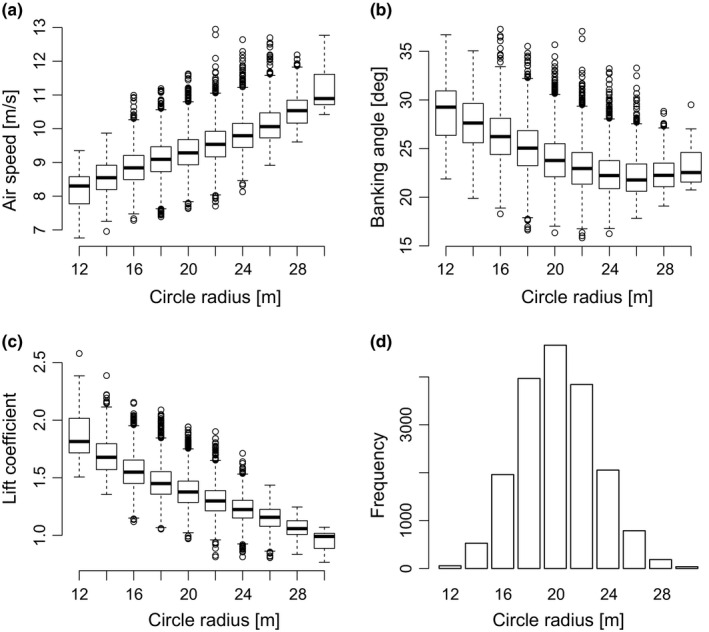
Circling behavior. (a) Storks flew faster in larger thermalling circles, which represent either larger thermals, or thermalling flight farther away from the centre of the thermal (intercept = 6.73 ± 0.04, slope = 0.13 ± 0.001, *n* = 18,073). (b) Smaller thermalling circles require larger banking angles, but intermediate and large thermalling circles do not affect the banking angle. (c) Lift coefficient is reduced in larger thermalling circles (intercept = 2.23 ± 0.01, slope = −0.042 ± 0.001, *n* = 18,073). (d) The distribution of thermalling circle radii throughout our dataset

Similarly, here we examined whether and how storks adjust their behavior when circling in differently sized thermals. We showed that in smaller circles storks flew slower, thereby reducing the need to bank, but they still banked more than in larger circles (Figure 6). In theory, such increased banking angle requires the bird to generate more lift (equations (14) and (15)), whereas the reduced flight speed tends to reduce lift (equation (16)). Thus, birds flying in smaller circles needed to increase the amount of lift created per squared airspeed substantially, that is, increase their lift coefficient, by, for example, changing wing posture or angle of attack. By being able to study the difference in upwind and downwind circling airspeed between individuals, we can increase our knowledge on interindividual variation in thermalling‐flight skill (Harel, Horvitz, et al., 2016). Such detailed information on variation in flight skills between individuals of different age, experience or origin is difficult to obtain but it is key for understanding life history decision, survival and population dynamics (Sergio et al. 2014, Rotics et al. ) or differences between individuals of allopatric species (Friedemann et al., 2016). It can also inform us about the flight strategies of individuals as they overcome large geographic barriers, such as the Himalaya Mountains (Sherub et al., 2016).

## 5 Conclusions

We provide a numerically robust approach for calculation of wind velocities from bird‐borne data collected from high‐resolution GPS tags. Our approach also quantifies the uncertainty in the approximation of the observed wind speed. We show that the bird's behavior, which hypothetically may bias our calculation, does not violate our assumptions, and is therefore not a source of consistent bias. The results from this analysis and similar bird‐borne wind measurements provide key information for understanding the birds' flight behavior during thermalling.

Despite the global availability of large datasets that provide direct and indirect observations about the environment (Dodge et al., 2013; Pettorelli, Safi, & Turner, 2014), measuring small‐scale environmental variables, and particularly those that change rapidly (e.g., wind, turbulence and their altitudinal profiles; the depth of the atmospheric boundary layer; and patterns of ocean currents, salinity, and sea‐ice cover), still proves a challenge, even more so in remote areas (Harris & Browning, 2013). Recent examples show that tracked animals can directly act as sensors of their surroundings by providing possibilities to estimate environmental conditions based on the animal's behavior using information from animal‐borne tags that record the animals' location, speed, and acceleration (Charrassin et al., 2008; Shamoun‐Baranes, Bouten, Camphuysen, & Baaij, 2011; Shannon, Young, Yates, Fuller, & Seegar, 2002a; Shannon et al., 2002b; Treep et al., 2016). By utilizing the ever‐increasing number of high‐resolution animal movement data, we may soon be able to monitor inaccessible areas or weather phenomena that are otherwise difficult to observe (Kays, Crofoot, Jetz, & Wikelski, 2015). Our study and analysis approach demonstrate that wild free‐flying birds can provide important high‐resolution data about the vertical structure of wind and uplift in the atmospheric boundary layer.

## Conflict of Interest

None declared.
